# Honey in the Prevention and Treatment of Infection in the CKD Population: A Narrative Review

**DOI:** 10.1155/2015/261425

**Published:** 2015-06-17

**Authors:** Anna Francis, Yeoungjee Cho, David W. Johnson

**Affiliations:** ^1^Department of Renal Medicine, Princess Alexandra Hospital, Brisbane, QLD 4102, Australia; ^2^University of Sydney, Sydney, NSW 2006, Australia; ^3^Centre for Kidney Disease Research, Translational Research Institute, University of Queensland, Brisbane, QLD 4102, Australia

## Abstract

Infection is a major cause of morbidity and mortality at all stages of chronic kidney disease (CKD). Multiresistant organisms are becoming increasingly common, particularly in the CKD population. Unfortunately, the rapid evolution of antibiotic resistance has not been mirrored by innovation in new antibiotic agents. Novel treatments are therefore urgently needed. Honey has garnered much interest due to its broad-spectrum antibacterial properties based on extensive experimental data. Unlike conventional antibiotics, honey has an added advantage as it appears to avoid inducing antimicrobial resistance in bacteria. This review discusses the potential mechanisms of action and role of honey in infection management in the general population, epidemiology and special challenges of infections in CKD populations, and the clinical trial evidence pertaining to the safety and efficacy of honey for the prevention and treatment of infections in CKD population.

## 1. Introduction

Infection is a major cause of morbidity at all stages of chronic kidney disease (CKD) and can directly contribute towards patient mortality [1]. CKD patients may be at an increased risk of infection due to various reasons, including background impairment in host immunity, or from devices such as central venous catheters or Tenckhoff catheters for the purposes of haemodialysis or peritoneal dialysis (PD), respectively. Therefore, infection prevention and management in the CKD population requires holistic care. Pharmacological and nonpharmacological approaches are critical for attaining optimal outcomes. Patient level nonpharmacological strategies include education on hygiene, prevention of skin breakdown, optimal glycaemic control, and nutrition [2]. Hospital level nonpharmacological interventions include policies on reducing device insertion (e.g., using arteriovenous fistulas for dialysis access rather than haemodialysis catheters), optimal device insertion techniques, hand washing, and auditing of infection rates [2, 3]. Pharmacological intervention centres on antimicrobial and antiseptic agents, as well as vaccination [4].

Whilst antibiotics remain the mainstay of modern practice for treatment of infection and, to a lesser extent, for prevention, their use is limited by the widespread emergence of antimicrobial resistance, which is one of the most pressing problems currently facing modern medicine. The last 40 years have seen rapid emergence of multidrug resistant organisms (MROs) with a concurrent decrease in new antibiotics reaching the market [5]. The rates of antibiotic-resistant bacteria are among the highest in dialysis patients [6, 7]. This has triggered appreciable research interest in alternative adjunct approaches to infection prevention and management.

One such promising alternative adjunctive approach is the use of topical honey. Honey has long been used as a traditional medicinal agent, as evidenced by reference to its therapeutic use in Sumerian tablets from 3000 B.C. [8], as well as in the Torah, Bible, and Koran [9]. Since the 1980s, honey has consistently been reported in the medical literature as having broad-spectrum antimicrobial properties, including activity against a wide range of MROs, making it a potentially very attractive agent for infection prophylaxis and therapy [10–12]. Moreover, honey has been reported to have immune modulating and anti-inflammatory properties whilst avoiding the risk of inducing antimicrobial resistance [11, 13].

This narrative review will discuss the potential mechanisms of action and role of honey in infection management in the general population, epidemiology and special challenges of infections in CKD populations, and the clinical trial evidence pertaining to the safety and efficacy of honey for the prevention and treatment of infections in CKD population.

## 2. The Role of Honey in Preventing and Treating Infection in the General Population

Honey has been used as a therapeutic agent to promote wound healing from ancient times [8]. The healing properties of honey have been attributed to its ability to maintain a moist wound environment to promote healing, a high viscosity to create a protective barrier to further prevent infection, and antibacterial activity [14, 15]. Preclinical studies have reported its immune-modulatory capacity by demonstrating ability to stimulate monocytes to secrete cytokines, such as tumour necrosis factor-alpha (TNF-*α*), recognised to play an important role in wound repair process [16, 17]. The antimicrobial property of honey has been attributed to multiple mechanisms, including its high osmolarity to resolve wound oedema [18], acidity, reactive oxygen species [19], content of hydrogen peroxide, and nonhydrogen peroxide components (e.g., phytochemical compounds such as methylglyoxal) [20, 21]. It has been shown to induce cell lysis [22] and to prevent biofilm formation [23]. In animal models, local application of honey into surgical wounds was associated with improved wound healing and injection of honey was associated with decreased cytokine release [24–27].

Unlike conventional antibiotics, honey has broad-spectrum antimicrobial activity against viruses, bacteria (including mycobacteria and MRO such as vancomycin-resistant enterococci (VRE) and methicillin-resistant* S. aureus* (MRSA)), and fungi [11, 19, 24, 28–32]. The effectiveness of honey against multidrug resistant strains of bacteria is promising, especially in renal patients in whom MRSA is one of the major causes of infections [33, 34]. More importantly, repetitive exposures to honey were not associated with antibiotic resistance in bacteria, as its activity does not target the growth of specific organisms but rather is a product of multiple mechanisms [13]. In addition, honey may potentially be a useful adjunctive therapy, in combination with various antibiotics, as it has been shown to exert synergistic effects [35]. This is very exciting in the new paradigm of antibiotic resistance with which modern health care is grappling.

However, just as there are differences in the antimicrobial spectrum in antibiotics, the antimicrobial properties of honey can vary, and importantly not all types of honey possess antibacterial effect [36]. The US Food and Drug Administration (FDA) and the Australian Therapeutic Goods Administration (TGA) are among world regulators that have certified the safety of medical honey. There are various licensed medical-grade honeys, which have been tested for antibacterial properties and screened for contaminating pesticides [37]. The most commonly used honey is Manuka honey, produced by bees imbibing the nectar of the native New Zealand Manuka bush. Manuka honey has increased antibacterial effect compared to some other types of honey [38] and has demonstrated antibacterial efficacy at dilutions of 15–30%, whilst most other honeys can only be minimally diluted (>80%) [20, 30, 39]. This is likely due to its peroxidase as well as nonperoxidase (i.e., methylglyoxal) activity [20]. Most other honeys primarily exert their antimicrobial effect through peroxidase activity only. For this reason, for medical indications, standardised antibacterial honey must be used rather than household honeys. Honey in the form of dressing has been utilised with variable frequency (1–3 times a day) [40–42], whereas only once daily application has been evaluated for infection prophylaxis of Tenckhoff catheters in peritoneal dialysis patients [43].

As aforementioned, honey has been documented to augment wound healing and treat infection [44]. Individual case reports and case series of up to 59 patients have reported favourable outcomes from treatments using honey for skin infections [22, 23, 40, 45–50], including those caused by drug-resistant strains, such as MRSA [51]. One case series of 16 paediatric oncology patients reported excellent wound infection results in children treated with IV antibiotics and honey dressings [42]. This is of particular interest given the well-documented immunosuppressed state of patients with CKD, in whom patients with diabetes mellitus make up a significant subgroup [52]. However, more high-quality clinical evidence is needed to ascertain the exact effectiveness of honey before recommending its wider implementation in the clinical setting.

The evidence pertaining to the use of honey in acute and chronic wounds was recently examined in a Cochrane meta-analysis and systematic review [53]. The study identified 26 eligible trials (3011 participants) and included two high-quality evidence trials in whom honey dressings appeared to be superior when compared to conventional dressings in patients with partial thickness burns (2 trials; *n* = 992; WMD −4.68 days, 95% CI −5.09 to −4.28). However, the benefit was not uniformly observed as its effect was unclear for venous leg ulcers (2 trials, *n* = 476, low quality evidence), minor acute wounds (3 trials, *n* = 213, very low quality evidence), diabetic foot ulcers (2 trials, *n* = 93, low quality evidence), and mixed chronic wounds (2 trials, *n* = 93, low quality evidence), largely due to suboptimal level of available evidence. In spite of low quality evidence (1 trial, *n* = 50), honey appeared to delay wound healing when compared to early excision and grafting in patients with partial and full thickness burns (WMD 13.6 days, 95% CI 9.82 to 17.38) [54]. The strength of the conclusion that could be drawn from the review was compromised by the heterogeneous nature of the patient populations and comparators, and generally low methodological quality of the available evidence. For instance, Ingle and colleagues reported comparable efficacy of honey compared to hydrogel in healing of shallow wound and abrasions in 82 patients [55]. Although the study did identify honey as a cost-effective treatment modality, there was an unclear risk in terms of allocation bias as well as risks of performance and detection biases due to a lack of blinding of participants and treating physicians, respectively.

In addition to faster healing of wounds treated with honey, Malik and colleagues have reported lower bacterial colonisation in patients treated using honey [41]. However, these results have not been consistently replicated in other studies [56, 57]. Furthermore, a higher treatment withdrawal rate in patients treated using honey dressing compared to usual care was reported in one study (16.6% versus 0%), largely due to ulcer site-related concerns, such as pain [56]. Other studies suggest honey would need to be applied more often compared to conventional alternatives, which could negatively impact patient satisfaction, compliance, and treatment success [58].

To date, most research into the medical uses of honey centres on skin infections. However, honey has also been reported to be effective in treating chronically infected open mastoid cavities [59], to decrease the risk of endophthalmitis after eye surgery [60], and to be useful as an adjuvant therapy for chronic rhinosinusitis [61]. Honey was not found to prevent mucositis in a study of 131 oncology patients taking Manuka honey or placebo [62]. Honey applied to damaged rat cornea was associated with faster healing and decreased cytokine expression [63].

It is important to note that honey is not without a risk profile. Honey can be contaminated with* Clostridium botulinum* if processed incorrectly. However, sterilisation with gamma-radiation alleviates this risk [64]. Very rarely, people can also be allergic to honey, with reported cases of anaphylaxis [65]. In addition, methylglyoxal, one of the key antimicrobial components of Manuka honey, has been demonstrated to exert a direct cytotoxic effect on diabetic wound healing [66]. Other concerning features of honey include its ability to promote oxidative stress from production of hydrogen peroxide, which at high levels can lead to release of oxygen free radicals precipitating protein degradation [67]. The amount of hydrogen peroxide produced by topical application of honey is variable [68].

## 3. Infection in CKD

People with CKD are 3-4 times more likely to sustain serious infections than the general population [69]. For those on dialysis, the mortality risk from sepsis increases up to 50 times for CKD patients and up to 20 times for kidney transplant recipients compared with the general population [70]. The most commonly described infections are bloodstream, skin, internal organ, and device-related infections.

Device-related infections constitute the single largest group of serious infections in the renal population and include exit site/tunnel tract infections and bacteraemia complicating haemodialysis catheters and peritonitis complicating peritoneal dialysis catheters [71, 72]. Haemodialysis catheter infections occur at a rate of 1–10/1000 catheter days and peritoneal catheter infections at one episode/seven to 200 patient months [73–75]. Device infection can occur in multiple ways, including inoculation of skin flora at the time of insertion and local invasion of bacteria through the exit site in the skin or from contamination during handling of the catheter, for example, when accessing the catheter for dialysis [2]. Infection represents a serious public health burden, with a single hospitalisation episode for bacteraemia estimated to cost around $USD 20,000 [76]. Similarly, a hospitalised PD peritonitis episode is estimated to cost approximately $USD 12,000 [77].

CKD patients are at increased risk of infection due to a combination of uraemia-associated suppressed immunity, coexisting morbidity (particularly diabetes mellitus), hypoalbuminemia, anaemia, iron overload, uraemia, and malnutrition [78–82]. These factors in turn engender impaired neutrophil phagocytosis and antigen processing by lymphocytes [79, 82, 83]. Dialysis itself increases infection risk, as does the immunosuppression required in kidney transplantation [82]. All of these risk factors occur in a population that requires frequent health care utilisation, which unfortunately further exposes them to bacterial pathogens.

The microbiology of infection in CKD varies with the site of infection [84]. In general, Gram-positive organisms such as* Staphylococcus* are the most prevalent, particularly for device-related infections [85]. For example, PD-related peritonitis is caused by Gram-positive bacterial infection in around half of cases (53.4%), with fewer cases due to Gram-negative organisms (23.6%) [84]. Similarly, a recent meta-analysis of 1596 patients with haemodialysis catheter-associated bacteraemia found* Staphylococcus aureus* to be responsible for 25.9% of cases,* Staphylococcus epidermidis* for 23.4%, and Gram-negative rods for 22% [86].

The emergence of multiresistant organisms in the CKD population is of growing concern. The first case of VRE occurred in a dialysis patient [87], likely a result of the frequent and widespread use of antibiotics in this population [88]. The large burden of infections, particularly due to MROs, has fuelled an urgent need to find alternative means of preventing and treating infections in CKD patients besides antibiotics use.

## 4. Effect of Honey in Preventing Infection in CKD Patients

Although honey has primarily been employed in Western medicine for the prevention of skin infections, there is emerging evidence that this agent may be particularly useful in CKD populations because of its very broad antimicrobial spectrum and lack of induction of antimicrobial resistance.

One of the earliest studies of honey as an infection prophylaxis agent in CKD patients was a single-centre, open-label, parallel-arm randomised controlled trial by our group of thrice-weekly exit site application of standardised antibacterial honey versus mupirocin on bloodstream infection rates in 101 haemodialysis patients with tunnelled, cuffed haemodialysis catheters [89]. This study found that catheter-associated bloodstream infection rates were not significantly different between the honey group (0.97 episodes per 1000 catheter days) and controls (0.85 episodes per 1000 catheter days). Following adjustment for age, sex, race, body mass index, diabetic status, ischaemic heart disease, presence of infection at the time of randomisation, nasal staphylococcal colonization, and serum albumin, honey administration resulted in comparable infection-free survival compared with standard mupirocin antibiotic prophylaxis (adjusted hazard ratio 0.94, 95% CI 0.27–3.24, *P* = 0.92). No exit site infections were observed in either group over the median (interquartile range) follow-up period of 95 (55–157) days. Importantly, 26 (2%) of 1328 staphylococcal isolates during the period of the trial were found to be mupirocin-resistant. Honey was well tolerated by patients and costs were similar between the honey and mupirocin antibiotic groups. Thus, honey was found to be safe, cheap, and effective for preventing haemodialysis catheter-associated infections. These findings together with those of previous studies demonstrating a very low likelihood of selecting resistant organisms led to recommendations for routine use of topical honey in the Queensland Infection Surveillance and Prevention Guidelines for Haemodialysis Catheters [90]. Since routinely converting from mupirocin to honey chemoprophylaxis in our haemodialysis unit, median infection-free catheter survival has remained excellent at 0.58 years (95% CI 0.31–0.85 years) with a fall in observed mupirocin-resistant staphylococcal isolates (~1%) [91].

The other study included in the Cochrane review was of 49 haemodialysis patients randomised to sterilised Manuka honey or povidone-iodine dressings applied to catheter exit sites after each dialysis session [92]. This study was only briefly reported in a letter to the editor. It is unclear whether some or all catheters were temporary catheters, which are well described to have an increased rate of bacteraemia [93]. The study found no difference in exit site infection or bacteraemia rates between the honey and povidone-iodine groups.

The previous two studies were collectively reviewed as part of a Cochrane review of interventions to prevent infectious complications of haemodialysis patients [94]. One section reviewed honey versus antimicrobial ointments for preventing catheter-associated infections. They found nonsignificant risk ratios of 0.45 (95% CI 0.1–2.11) for honey preventing exit site infection and 0.8 (95% CI 0.37–1.73) for honey preventing catheter-related bacteraemia. This review was limited by the small sample size (*n* = 150), the granularity of the data, and the heterogeneity of the two studies.

Honey has also been evaluated in peritoneal dialysis patients. Our group published the largest study in this area to date, the HONEYPOT trial, in 2014 [43]. Three hundred and seventy-one patients on peritoneal dialysis were randomised 1 : 1 to daily honey application to the Tenckhoff catheter exit site or to standard care (nasal application of mupirocin to staphylococcal carriers). All other care methods, including exit site management, were identical between the groups. No difference in time to first catheter-associated infection (exit site infection, tunnel infection, or peritonitis) was observed between the two groups (unadjusted hazard ratio 0.12, 95% CI 0.83–1.51, *P* = 0.47). In a prespecified subgroup analysis of diabetics, honey application resulted in significantly shorter times to first infection (HR 1.85, 95% CI 1.05–3.24) and peritonitis (HR 2.25, 95% CI 1.16–4.36) compared with controls, suggesting that honey may have been an inferior infection prevention agent in this group of patients. Interestingly, mupirocin-resistant staphylococcal isolates were observed in the control group but not in the honey group, although the numbers were too small to permit meaningful analysis. Whilst serious adverse events were comparable between the honey and control groups (298 versus 327, resp., *P* = 0.2), 11 (6%) patients in the honey group experienced local skin reactions to honey and 54/186 (29%) of the honey group withdrew from the study compared to 17/185 (9%) of the control group. The relatively high withdrawal rate and possible inferior results in diabetics argued against a role for routine use of honey as a chemoprophylactic agent for the prevention of peritoneal dialysis catheter-associated infections.

## 5. Effect of Honey on Treating Infection in Chronic Kidney Disease

Although there have been a number of studies evaluating the safety and efficacy of honey in the treatment of skin and wound infections in patients at risk of CKD, such as diabetics [44, 95], there have been no clinical or preclinical studies of the use of this agent in patients with CKD* per se*. In a rat model of bacterial peritonitis following caecal ligation and puncture, Yuzbasioglu et al. demonstrated that intraperitoneal administration of honey resulted in lower peritoneal adhesion scores and tissue oxidative stress levels at day 14 compared with rats receiving intraperitoneal 5% dextrose or no treatment at all [96]. This raised the interesting possibility that honey may have a potential useful adjunctive role in the management of PD-associated peritonitis, although the study was limited by its small sample size, lack of blinding of outcome assessors, and uncertain generalizability of the model to the clinical scenario of PD-associated peritonitis. Clearly, further studies of the therapeutic efficacy of honey in infected CKD patients are warranted.

## 6. Summary and Future Directions

Honey is an appealing addition to our weaponry against infections due to its broad antimicrobial effect without inducing resistance [15]. These properties are particularly attractive in CKD patients in whom infections, including those caused by MROs, are more prevalent due to background immunodeficiency state, increased frequency of device insertion, and health care utilization [1].

Whilst there is much experimental evidence to support the biological plausibility of honey as an effective therapeutic agent [11], data from clinical trials, predominantly studied in the areas of wound infection or management, have been inconsistent [38, 48, 55, 56, 89, 95, 97, 98]. There is also a paucity of large randomised controlled trials examining the effectiveness of honey as both a prophylactic and therapeutic agent for infection. Moreover, the limited data so far available are difficult to interpret due to small sample sizes and generally suboptimal methodological quality. It may be that honey has limited applicability in subgroups like patients with diabetes mellitus. Some studies also highlight the issue of compliance with interventions using honey, with high treatment withdrawal rate (e.g., 29%) [43]. The cost-effectiveness of honey is also difficult to ascertain with contrasting reported outcomes [55, 56].

Studies are required to better evaluate the causes for variable compliance in interventions using honey to help improve adherence (e.g., developing new preparations to mitigate skin reactions). Furthermore, adequately powered and well-designed future studies into honey are warranted, in both treating and preventing infections in CKD (e.g., prevention of infection in postdialysis access operation wounds) and general populations (e.g., applicability of honey dressings for treating foot ulcers).
